# Tall fescue endophyte effects on tolerance to water-deficit stress

**DOI:** 10.1186/1471-2229-13-127

**Published:** 2013-09-09

**Authors:** Padmaja Nagabhyru, Randy D Dinkins, Constance L Wood, Charles W Bacon, Christopher L Schardl

**Affiliations:** 1 Department of Plant Pathology, University of Kentucky, Lexington, KY 40546-0312, USA; 2 USDA-ARS, Forage-Animal Production Research Unit, Lexington, KY 40546-0091, USA; 3 Department of Statistics, University of Kentucky, Lexington, KY 40506-0027, USA; 4 USDA-ARS, Toxicology and Mycotoxin Research Unit, Athens, GA 30605-2720, USA

**Keywords:** Fungal endophyte, Tall fescue, Water deficit stress, Metabolites, Neutral sugars, Amino acids and lolines

## Abstract

**Background:**

The endophytic fungus, *Neotyphodium coenophialum*, can enhance drought tolerance of its host grass, tall fescue. To investigate endophyte effects on plant responses to acute water deficit stress, we did comprehensive profiling of plant metabolite levels in both shoot and root tissues of genetically identical clone pairs of tall fescue with endophyte (E+) and without endophyte (E-) in response to direct water deficit stress. The E- clones were generated by treating E+ plants with fungicide and selectively propagating single tillers. In time course studies on the E+ and E- clones, water was withheld from 0 to 5 days, during which levels of free sugars, sugar alcohols, and amino acids were determined, as were levels of some major fungal metabolites.

**Results:**

After 2–3 days of withholding water, survival and tillering of re-watered plants was significantly greater for E+ than E- clones. Within two to three days of withholding water, significant endophyte effects on metabolites manifested as higher levels of free glucose, fructose, trehalose, sugar alcohols, proline and glutamic acid in shoots and roots. The fungal metabolites, mannitol and loline alkaloids, also significantly increased with water deficit.

**Conclusions:**

Our results suggest that symbiotic *N. coenophialum* aids in survival and recovery of tall fescue plants from water deficit, and acts in part by inducing rapid accumulation of these compatible solutes soon after imposition of stress.

## Background

Tall fescue (*Lolium arundinaceum = Schedonorus arundinaceus* = *Festuca arundinacea*) is the most widely planted forage grass in the United States [1] and it is often infected with the endophytic fungus, *Neotyphodium coenophialum.* The relationship between the endophyte and plant is generally considered mutualistic because the endophyte significantly improves host plant tolerance to drought, insects, diseases, and nematodes, along with increased persistence and vigor; and in turn the plant provides the symbiont with nutrients, protection, and reliable and efficient dissemination (reviewed in [2]). Evidence suggests that tall fescue plants with the endophyte (E+) grow and persist longer under stressful conditions, such as water deficit, compared to endophyte free plants (E-), and are, therefore, likely to have an adaptive and competitive advantage [3-9]. Mechanisms for endophyte-enhanced drought avoidance or tolerance appear complex, and might involve direct and indirect effects of the endophyte on metabolism and other physiological changes in the host plant [10-13].

Processes affected by the tall fescue endophyte include stomatal closure [14], decreased root diameter and increased root hair length [7,15], increased turgid weight/dry weight ratios suggesting reduced damaged to cell walls [10], and enhanced production of phenolic root exudates [15]. Leaf rolling under drought stress is reported to be much more common in E+ than E- plants [3]. Greater cell wall elasticity [10] and higher water use efficiency [16] in E+ tall fescue compared to E- plants under drought stress have also been reported. Previous research has also shown that E+ tall fescue plants of some genotypes exhibit lower stomatal conductance than E- plants with more sensitive inducement of stomatal closure in E+ plants in response to early stages of water deficit [17-19]. Endophyte infection confers population stability in tall fescue during drought stress through improved tiller and whole plant survival [5].

A correlation between drought tolerance and accumulation of compatible solutes such as carbohydrates, amino acids, and mineral ions that contribute to osmotic adjustment has been documented in grasses [20-22]. In general, accumulation of sugars, sugar alcohols [23], and proline [24,25] in response to water deficit in grasses has been reported. A significant endophyte effect on accumulation of simple sugars in leaves of E+ tall fescue, was observed when plants were osmotically stressed by polyethylene glycol [26]. Under water deficit, E+ tall fescue plants are reported to exhibit decreased growth and increased root and leaf senescence, as well as greater accumulation of sugars within the pseudostem, and decreased water potential compared to E- plants [27]. Effects of the endophyte on levels of other metabolites, such as proline [28] and other amino acids have not been well studied. Here we report what is, to our knowledge, the first comprehensive profiling of shoot and root metabolite responses to acute water deficit stress, assessing the timing of endophyte effects on sugars, sugar alcohols and amino acids relative to the endophyte effects on subsequent plant recovery.

## Methods

### Experimental design

Tall fescue is an obligately outcrossing grass, so that isogenic lines cannot be generated, and plants derived from different seeds are necessarily unique genotypes. Therefore, to control for host genotype effects we developed genetically identical clones with endophyte (E+) and without endophyte (E-) as follows. Ramets of tall fescue ‘Kentucky 31’ plants naturally infected with *Neotyphodium coenophialum* were treated with the fungicide propiconazole or tebuconazole to remove the fungus [29,30]. The stock plants and fungicide-treated clones were examined for the presence or absence of endophyte by tissue print immunoblot [31], PCR [32], and microscopy. This resulted in E+/E- clone pairs, two of which were used in this study. Lab identification numbers 278 (E+) and 279 (E-) represented one clone pair, and 4607 (E+) and 4608 (E-) represented the other clone pair. Plants of each clone pair were raised side-by-side in the greenhouse for more than one year prior to being used in the study.

Ramets consisting of three tillers of similar size were planted into 8.5 × 8.5 cm square pots in sand, in the greenhouse. Sand was chosen as the growth medium because it allows even, uniform and rapid drying, and also provides for easy harvesting of roots. Plants were watered twice daily for six weeks before subjecting them to experimental conditions to allow for regeneration and accumulation of sufficient biomass for sampling. After sufficient re-growth had occurred, water was withheld from the test group, while control plants were watered twice daily. Pots were randomized once while setting up the experiment and again before subjecting them to treatments, in order to control for effects micro-environmental variation.

Treatments were endophyte-infected watered controls (E+D-), endophyte-infected water-deficit stressed (E+D+), endophyte-free watered controls (E-D-), and endophyte-free water-deficit stressed (E-D+). Entire pots were sampled on each day from day 0 to day 5 of withholding water. Beyond day 5 plants were fully dried and mostly dead. Three or four replicates were sampled for each treatment x day. For the first experiment, which was conducted with the 278/279 clone pair, samples were harvested from February 2–7, 2007. For the second experiment with clone pair 278/279, samples were harvested from June 2–7, 2008. The third experiment was conduced with clone pair 4607/4608, sampled from July 21–26, 2008. All plants were grown in the greenhouse under natural light conditions, with 45-70% relative humidity ranges, and temperatures set to 27°C/22°C (day/night). Photoactive radiation (PAR) measurements were recorded during the three experiments (see Additional file 1, panels a, b, c). Samples were harvested between 7:30 a.m. to 8:30 a.m. local time each day, immediately frozen in liquid nitrogen, lyophilized and subsequently prepared for metabolite analysis as described below. The samples were divided into shoot (leaf along with tiller base down to 1 cm from crown region) and root material.

### Tiller recovery experiment

Five to six pots subjected to water-deficit conditions from each E+/E- clone pair for each day of treatment were left unharvested, and were placed back into a daily watering regime in order to determine their ability to recover from the water-deficit stress. Live tiller numbers were counted after 6 weeks of recovery.

### Carbohydrate analysis by high pH anion exchange chromatography

Sugars were extracted in 1 ml of 80% ethanol per 100 mg of ground lyophilized plant material. The samples were incubated at 65°C for 1 hr and 90°C for 5 min and the supernatant was evaporated in a vacuum centrifuge. The residue was reconstituted in purified water at 4°C and filtered through spin-X HPLC 0.4 μm nylon filter micro centrifuge (Corning, NY) tubes. Filtered supernatant (100 μL) was diluted to 1 ml and used for analysis on a Dionex ICS 3000 with either a carbopac PA1 column for neutral sugars or a carbopac MA1 column for polyols. Neutral sugars were separated by an isocratic program with 24 mM NaOH, and sugar alcohols were separated using 480 mM NaOH. The detection was by pulsed amperometry, using a gold working electrode. Peak identity and sugar quantity were determined by comparison with standards. The internal standard was 2-deoxyglucose.

### Amino acid analysis by liquid chromatography-mass spectrometry (LC-MS)

The yields of free amino acids from plant samples were compared for different extraction methods using a) 80% ethanol, or b) chloroform: methanol: water (5:12:3), and incubating at different temperatures (4°C and 45°C) for 1 hr. However both extraction solvents and methods resulted in similar extraction efficiency, so the simpler extraction method was chosen for further analysis. Finely ground lyophilized plant shoot and root material (50 mg) was extracted with 5 ml of 80% ethanol on ice for 1 hr. The crude extract was filtered through 0.4 μm centrifuge tubes and the supernatant was used for sample cleanup and derivatization with EZ faast LCMS kit for free amino acids from Phenomenex, according to the kit protocol. Briefly, 100 μL of each sample was mixed with 100 μL of internal standard containing homoarginine, d3-methionine, and homophenylalanine provided in the kit. Then sample was loaded onto a pipet tip packed with ion exchange resin on which free amino acids were bound, subsequently washed and released from resin. The free amino acids were then derivatized by propyl chloroformate and liquid-liquid extracted with isooctane. The organic phase containing the derivatized amino acids was removed under a stream of high purity nitrogen gas and the residue was redissolved in 200 μL 2:1 mobile phase of A:B (A: 10 mM ammonium formate in water and B: 10 mM ammonium formate in methanol). Analysis was performed by liquid chromatography mass spectrometry with a dual pump ProStar 210 HPLC with 1200 L quadrupole MS-MS (Varian).

### Loline alkaloid analysis

Loline alkaloids were extracted from samples using chloroform under alkaline conditions [33]. Quinoline was used as an internal standard and the lolines were quantified by gas chromatography (Varian CP-3800) interfaced with a Varian Saturn 2200 ion trap mass spectrometer. Loline amounts were calculated as the total of loline, *N*-methylloline, *N*-formylloline, *N*-acetylloline and *N*-acetylnorloline.

### Statistical analysis

Factorial Analysis of Variance (2 × 2 × 6) was run to analyze tiller recovery and metabolite levels in PROC GLM, SAS (SAS Institute Inc., Cary, NC, USA.). Following ANOVA, tiller recovery and metabolite levels of E+ and E- clones were compared on each day using Estimate Statements. In order to control the overall α-level for multiple tests, the distribution of the maximum of the absolute value of elements of a multivariate (six variate) t-distribution with **μ** = **0** and ∑ = **I**[34], i.e. t-max, was used to calculate the significance levels for each of the six t-tests. Because of the extremely conservative nature of this procedure, α = 0.10 is used to determine significance of differences [p. 71 in ref. [35]. The three factor ANOVAs of all metabolites in all three experiments are given in Tables 1, 2, and 3. For the tiller numbers after recovery, four biological replications were run for each treatment in the first experiment on clone pair 278/279, and five biological replications were run for the other two experiments on clone pair 278/279 and clone pair 4607/4608. For metabolites, four biological replications were run for each treatment in the first experiment, and three biological replications were run for the other two experiments. In the graphs the significant differences of various metabolites between E+ and E- plants in the water deficit stress treatments were represented based on t-max values; ‘*’ denotes *p*- values = > 0.01 - 0.10; ‘**’ denotes *p*-values > 0.001 - 0.01; ‘***’ denotes *p*- values < 0.001.

**Table 1:** **Three-factor ANOVA [F**_**df**__**(5,72)**_**] values of all metabolites in Experiment 1**

**Metabolite**	**Endophyte**	**Day**	**Stress**	Day * Endophyte	Stress * Endophyte	Stress * Day	Endophyte * Stress * Day
Shoot glucose	48.89***	54.96 ***	36.91***	6.05***	2.37	7.13***	39.52***
Shoot fructose	6.77*	35.59 ***	143.32***	4.64***	0.88	9.37***	8.75***
Shoot sucrose	13.18***	13.97***	113.23***	2.38	0.23	15.59***	2.58**
Shoot GFS	1.72	37.69***	206.12***	0.96	0	9.72***	9.85***
Shoot proline	0.2	148.29***	178.23***	12.33***	4.50**	145.27***	16.86***
Shoot glutamine	0.03	26.92 ***	33.29***	3.61**	0.26	6.16***	3.24**
Shoot glutamic acid	6.55*	64.80***	61.02***	3.56**	11.03***	9.48***	5.88***
Shoot asparagine	0.8	8.27***	25.16***	2.11	0.24	8.49***	2.70*
Shoot aspartic acid	0.98	19.41***	0.49	3.37**	0.14	10.01***	10.11***
Shoot tryptophan	20.09***	40.29***	533.71***	2.60*	0.02	41.42***	9.05***
Shoot phenylalanine	9.94**	78.52***	564.58***	7.90***	36.25***	56.47***	17.66***
Shoot tyrosine	34.61***	19.69***	67.21***	9.91***	5.33*	15.41***	9.73***
Shoot lolines	1930.18***	3.11*	17.84***	3.11*	17.84***	9.97***	9.97***
Root glucose	0.4	17.57***	0.38	10.03***	7.12***	6.16***	14.24***
Root fructose	0.2	27.72 ***	22.17***	5.39***	4.93*	18.99***	15.73***
Root sucrose	0.02	9.19 ***	36.32***	1.97	0.02	8.71***	4.75***
Root GFS	0.13	12.59***	25.81***	5.22***	4.10*	5.80***	6.04***
Root proline	1.37	16.07 ***	209.87***	7.74***	0.17	18.11***	6.19***
Root glutamine	5.51*	29.58***	27.53***	18.69***	3.17	6.67***	1.35
Root glutamic acid	7.13**	50.81***	86.53***	23.01***	0	5.57***	14.41***
Root asparagine	40.63***	5.21***	43.23***	3.09*	0.75	2.87*	1.7
Root aspartic acid	5.55*	32.52***	18.15***	7.71***	5.69*	10.40***	10.25***
Root tryptophan	6.35*	4.88***	0.23	1.75	0.63	2.14	3.40*
Root phenylalanine	18.44***	20.94***	8.01**	9.37***	10.26**	7.83***	3.00*
Root tyrosine	13.03***	7.95***	0.03	4.07**	8.11**	2.64*	2.32
Root lolines	1914.28***	3.64**	35.14***	3.64**	35.14***	1.64	1.64

‘*’ denotes *p*-values = > 0.01 - 0.05; ‘**’ denotes *p-*values = > 0.001 - 0.01; ‘***’ denotes *p*-values = < 0.001.

**Table 2:** **Three-factor ANOVA [F**_**df**__**(5,48)**_**] values of all metabolites in Experiment 2**

**Metabolite**	**Endophyte**	**Day**	**Stress**	Day * Endophyte	Stress * Endophyte	Stress * Day	Endophyte * Stress * Day
Shoot glucose	42.30***	11.03 ***	15.13***	0.61	1.84	2.93*	3.08*
Shoot fructose	39.02***	14.48 ***	21.23***	1.45	2.6	3.69**	3.35**
Shoot sucrose	17.26***	16.55***	0.01	2.33	0.12	3.51**	0.68
Shoot GFS	37.84***	15.85***	8.37**	0.67	1.38	2.98*	1.67
Shoot proline	25.90***	124.48***	581.24***	7.46***	25.52**	126.22***	7.31***
Shoot glutamine	11.43**	42.89 ***	113.19***	2.39*	0.05	28.25***	1.09
Shoot glutamic acid	0	7.19***	34.68***	0.4	0.44	6.66***	0.34
Shoot asparagine	10.25**	12.48***	79.38***	1.05	1.16	18.03***	0.38
Shoot aspartic acid	2.37	7.66***	6.45*	1.47	0.12	5.49***	0.22
Shoot threonine	0.19	48.84***	260.74***	1.97	3.46	58.15***	0.95
Shoot tryptophan	0.88	80.90***	245.37***	0.61	2.66	60.11***	0.75
Shoot phenylalanine	0.27	91.52***	431.11***	0.12	1.4	88.98***	0.31
Shoot tyrosine	17.32***	18.31***	204.79***	3.35*	2.16	38.27***	0.92
Shoot lolines	486.57***	2.21	15.81***	2.21	15.81***	3.05*	3.05*
Shoot mannitol	89.37***	19.68***	18.41***	13.54***	16.47***	4.22**	3.84**
Shoot arabitol	15.72***	6.25***	26.29***	3.26**	21.25***	5.66***	3.77***
Shoot sorbitol	10.36**	4.01**	3.67	0.85	1.48	1.87	1
Shoot myo-inositol	4.98*	17.81***	14.50***	1.3	0.66	4.28**	1.2
Shoot trehalose	13.11***	21.50***	5.65*	0.71	7.38**	7.35***	0.8
Root glucose	28.46***	5.10***	20.44***	2.61*	5.14*	3.97**	2.33*
Root fructose	37.16***	23.23 ***	11.34***	4.24**	29.11***	1.77	1.85
Root sucrose	5.67*	6.50 ***	28.68***	5.83***	5.99*	3.86**	2.36*
Root GFS	11.09**	14.02***	0.05	3.90**	20.57***	2.03	2.92*
Root proline	46.51***	28.74 ***	121.16***	10.55***	43.90***	29.80***	11.07***
Root glutamine	3.18	10.28***	38.96***	1.26	0.61	8.63***	1.15
Root glutamic acid	19.29***	9.22***	0.12	4.34**	1.7	0.46	0.3
Root asparagine	17.03***	14.58***	0.28	1.91	3.84	2.24	1.99
Root aspartic acid	32	7.86***	1.72	4.02**	0.84	3.31*	2.19
Root threonine	0	19.91***	93.19***	7.36***	13.08***	19.69***	2.46*
Root phenylalanine	78.70***	90.43***	177.24***	96.78***	100.52***	98.98***	80.49***
Root lolines	249.61***	0.73	36.01***	0.73	36.01***	3.14*	3.14*

‘*’ denotes *p*-values = > 0.01 - 0.05; ‘**’ denotes *p-*values = > 0.001 - 0.01; ‘***’ denotes *p*-values = < 0.001.

**Table 3:** **Three-factor ANOVA [F**_**df (5,48)**_**] values of all metabolites in Experiment 3**

**Metabolite**	**Endophyte**	**Day**	**Stress**	Day * Endophyte	Stress * Endophyte	Stress * Day	Endophyte * Stress * Day
Shoot glucose	0.55	15.38 ***	188.58***	1.7	0.18	30.31***	2.67*
Shoot fructose	5.01*	24.51***	344.35***	1.49	11.72	34.71***	1.56
Shoot sucrose	4.78*	26.49***	97.38***	0.39	5.94*	17.83***	2.98*
Shoot GFS	3.27	24.76***	320.43***	1.15	7.24**	35.35***	2.01
Shoot proline	0.5	37.81***	329.02***	0.87	0.6	38.41***	0.81
Shoot glutamine	0.01	9.91 ***	117.18***	1.94	0.02	12.05***	2.62*
Shoot glutamic acid	3.83	7.29***	0.66	3.64**	1.97	6.94***	2.67*
Shoot asparagine	0.82	8.43***	30.99***	0.41	0.12	3.87**	1.13
Shoot aspartic acid	0.76	1.17	33.33***	1.37	0.33	3.33*	0.52
Shoot tryptophan	5.01*	24.51***	344.35***	1.49	11.72**	34.71***	1.56
Shoot phenyl alanine	4.78*	26.49***	97.38***	0.39	5.94*	17.83***	2.98*
Shoot tyrosine	3.27	24.76***	320.43***	1.15	7.24**	35.35***	2.01
Shoot threonine	0.55	15.38***	188.58***	1.7	0.18	30.31***	2.67*
Shoot trehalose	23.82***	10.87***	69.51***	2.83*	11.06**	16.26***	5.79***
Shoot lolines	507.88***	2.35	18.51***	2.35	18.51***	1.23	1.23
Root glucose	0	3.41*	2.54	0.95	0.55	4.62**	1.87
Root fructose	2.2	8.00 ***	103.20***	0.93	1.56	14.24***	2.75*
Root sucrose	4.23*	13.00***	32.14***	2.05	1.98	9.98***	1.27
Root GFS	3.22	11.21***	72.15***	1.43	1.44	13.96***	2.51*
Root proline	0	9.55***	59.35***	1.93	0	8.94***	1.67
Root glutamine	1.95	3.22*	24.14***	2.06	0.73	2.18	1.55
Root glutamic acid	0.38	12.94***	6.74*	0.56	0.05	0.56	0.54
Root asparagine	1.11	2.39	4.28*	1.21	1.61	0.4	1.53
Root aspartic acid	0.17	32.52***	18.15***	0.47	0.08	3.75**	0.34
Root tryptophan	0.25	5.62***	56.82***	1	0.03	7.29***	0.46
Root phenylalanine	0.31	5.38***	54.78***	1.13	0.03	7.01***	0.91
Root tyrosine	0.42	1.35	13.37***	0.9	0.13	3.97**	0.77
Root threonine	2.07	7.32***	84.63***	1.13	2.75	9.03***	1.37
Root lolines	455.13***	9.26***	19.28***	9.26***	19.28***	0.93	0.93

‘*’ denotes *p*-values = > 0.01 - 0.05; ‘**’ denotes *p-*values = > 0.001 - 0.01; ‘***’ denotes *p*-values = < 0.001.

## Results

### Tiller number and recovery

**Figure 1:**
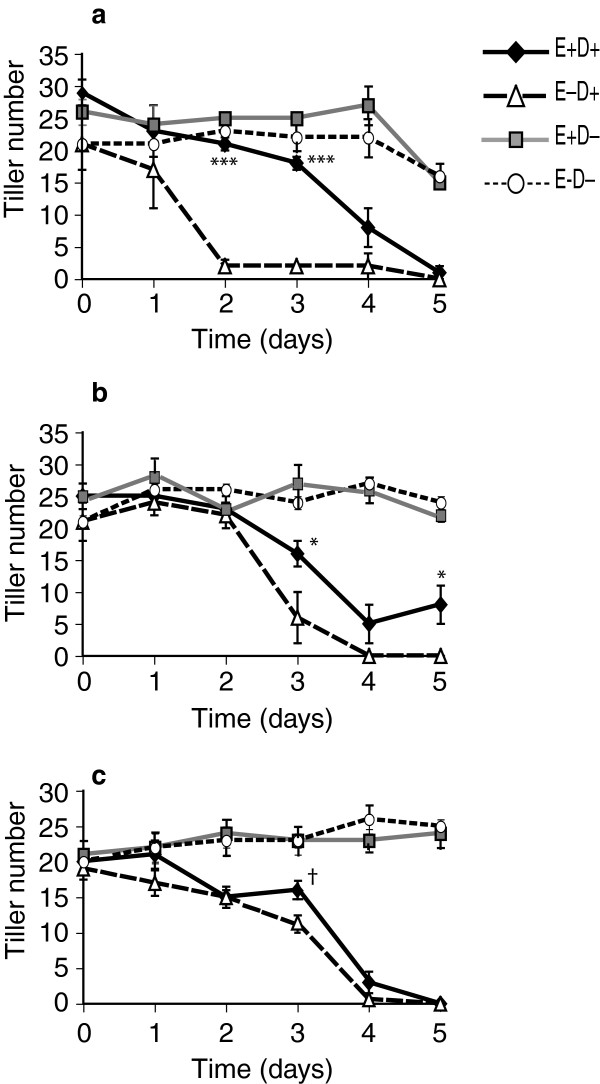
**Tiller recovery from water-deficit stressed E+ and E- plants after placing them back into normal watering regime. (a)** First clone pair 278 (E+)/279 (E-), Experiment 1, error bars are SEM (n = 4); **(b)** First clone pair 278 (E+)/279 (E-), Experiment 2, error bars are SEM (n = 5); **(c)** Second clone pair 4607(E+)/4608 (E-), error bars are SEM (n = 5). E+ D+ = endophyte infected and water withheld for the time periods indicated; E-D + = endophyte uninfected and water withheld; E+ D- = endophyte infected and unstressed; E-D- = endophyte uninfected and unstressed. Symbols indicating statistical significance based on t-max are ‘***’ *p* < 0.001; ‘**’ *p* > 0.001 - 0.01; ‘*’*p* > 0.01 - 0.1; ‘†’ *p* = 0.110 based on t-max; *p* = 0.019 based on t values).

Overall the E+ plants survived the stress conditions imposed during the experiment better than the E- plants when number of tillers produced upon recovery was used as the measure. In the first experiment with clone pair 278/279, after 2–4 days of withholding water, E+ plants produced more tillers than E- plants during recovery. However, after 5 days withholding water, none of the E+ or E- plants recovered (Figure 1a). In the second experiment with the same clone pair, starting at 3 days of withholding water, tiller recovery was significantly higher in E+ clones (Figure 1b). With clone pair 4607/4608 (Figure 1c), after 3-days of water deficit there was greater tillering of E+ plants, which was marginally significant (*p =* 0.110 based on t_max_, *p =* 0.019 based on t values).

### Neutral sugars

**Figure 2:**
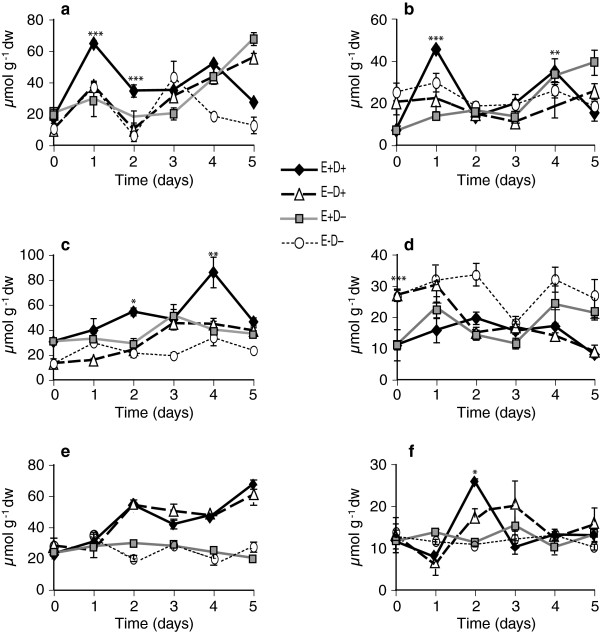
**Comparison of glucose levels in water-deficit stressed and unstressed shoots and roots of tall fescue. (a** and **b)** Shoots and roots, respectively, of the 278/279 clone pair, Experiment 1, error bars are SEM (n = 4); **(c** and **d)** shoots and roots, respectively, of the 278/279 clone pair, Experiment 2, error bars are SEM (n = 3); **(e** and **f)** shoots and roots, respectively, of the 4607/4608 clone pair, error bars are SEM (n = 3). E+ D+ = endophyte infected and water withheld for the time periods indicated; E-D + = endophyte uninfected and water withheld; E+ D- = endophyte infected and unstressed; E-D- = endophyte uninfected and unstressed. Statistical significance is indicated as in Figure 1.

**Figure 3:**
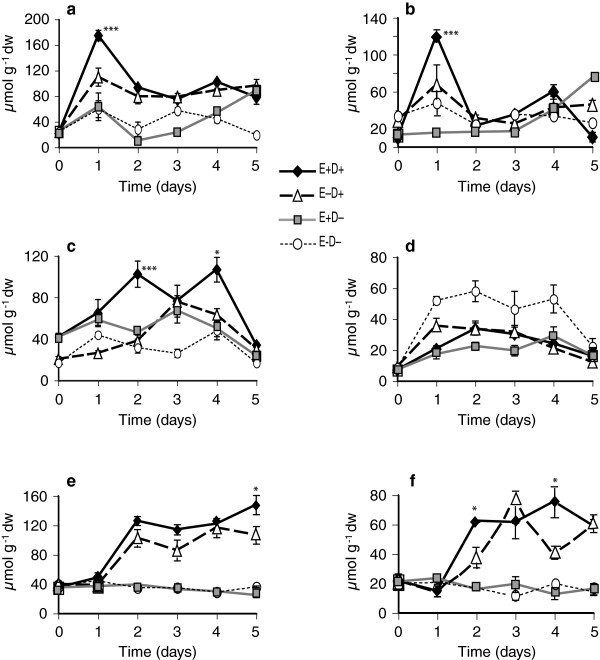
**Fructose levels in water-deficit stressed and unstressed shoots (a, c, and e) and roots (b, d, and f) of tall fescue. (a** and **b)** Shoots and roots, respectively, of 278/279 clone pair, Experiment 1; **(c** and **d)** shoots and roots, respectively, of 278/279 clone pair, Experiment 2; **(e** and **f)** shoots and roots, respectively, of 4607/4608 clone pair. Abbreviations are as in Figure 2. Statistical significance is indicated as in Figure 1. Error bars are SEM of biological replicates as indicated in Figure 2.

**Figure 4:**
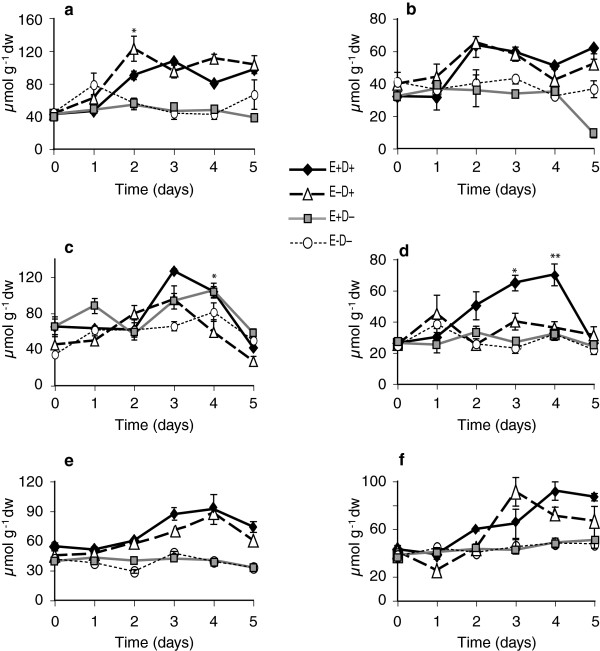
**Sucrose levels in water-deficit stressed and unstressed shoots and roots of tall fescue. (a** and **b)** Shoots and roots, respectively, of 278/279 clone pair, Experiment 1; **(c** and **d)** shoots and roots, respectively, of 278/279 clone pair, Experiment 2; **(e** and **f)** shoots and roots, respectively, of 4607/4608 clone pair. Abbreviations are as in Figure 2. Statistical significance is indicated as in Figure 1. Error bars are SEM of biological replicates as indicated in Figure 2.

The levels of galactose, glucose, fructose, sucrose, raffinose, stachyose, and trehalose were quantified in the tall fescue clone pairs in response to water deficit stress and endophyte infection. Of these, glucose, fructose and sucrose were the major free sugars identified. In Experiment 1 with clone pair 278/279, E+ shoots accumulated approximately 2-fold more free glucose and free fructose at day 1 compared to E- shoots (Figures 2a and 3a). Similarly in roots, free glucose and free fructose levels in E+ clones at day 1 after withholding water were significantly higher than in the E- clones (Figures 2b and 3b). In contrast, at day 1, E+ and E- clones showed no difference in sucrose levels compared to watered controls. Sucrose levels increased in shoots and roots of both E+ and E- clones starting from day 2 after withholding water (Figure 4a and b, Table 1). Comparing combined totals of glucose, fructose and sucrose at day 1, E+ clones had approximately 2–4 fold higher levels in shoots and roots compared to their watered controls, whereas the totals in E- clones did not differ significantly from their watered controls (see Additional file 2, panels a, b).

Results in Experiment 2, also with clone pair 278/279, were very similar except for a one-day delay in effects on tiller survival and metabolites, probably because of overcast skies on the first day (see Additional file 1, panel b). At day 2 of withholding water, free glucose and fructose levels in E+ were approximately 2–4 fold higher than in watered controls or in E- stressed plants (Figures 2c and 3c). There were no significant differences in sucrose levels at day 2 between E+ and E- plants (Figure 4c, Table 2). In roots, sucrose levels were 2–3 fold higher in E+ compared to E- roots from day 2 to day 4 (Figure 4d), though there were no significant differences in glucose or fructose (Figures 2d and 3d, Table 2, and see Additional file 2, panels c, d).

Comparing free glucose and fructose sugars in clone pair 4607/4608 during the water deficit period, there were significant differences between E+ and E- in the roots (Figures 2f and 3f); but not in shoots except for fructose at day 5, where E+ shoots accumulated fructose to higher levels than E- shoots (Figures 2e and 3e). Root glucose and fructose concentrations increased by day 2 of withholding water, and were significantly higher in E+ than E- plants.

**Figure 5:**
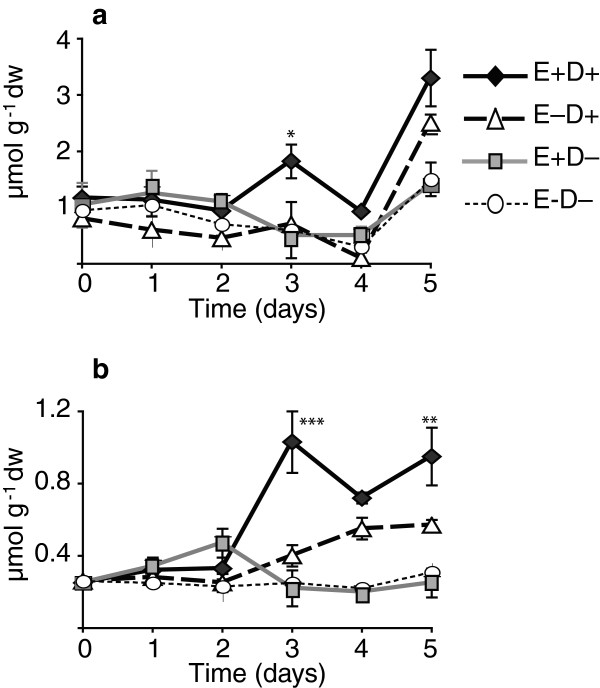
**Trehalose levels in shoot. (a)** 278/279 clone pair, Experiment 2; **(b)** 4607/4608 clone pair. Abbreviations are as in Figure 2. Statistical significance is indicated as in Figure 1. Error bars are SEM (n = 3).

The level of the disaccharide, trehalose, was low in the tall fescue clone pairs. However the trehalose levels were higher in water deficit tissues compared to the watered control samples (Tables 2 and 3), and after 3 days of withholding water significant higher levels of trehalose were observed in the E+ clones compared to the E- clones (Figure 5a and b).

### Sugar alcohols/polyols

**Figure 6:**
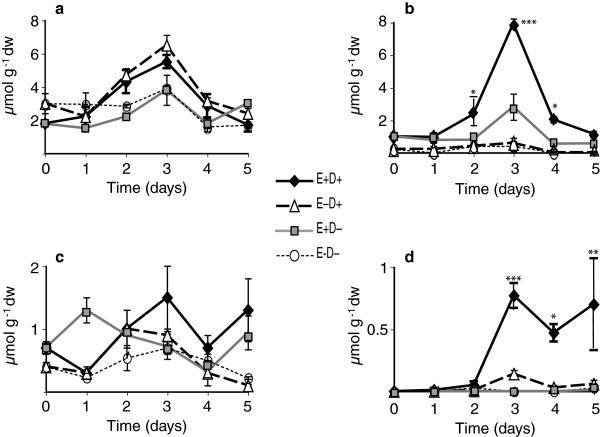
**Sugar alcohols or polyols in water-deficit stressed and unstressed shoots of tall fescue.** Shown are, **(a)** myo-inositol, **(b)** mannitol, **(c)** sorbitol, **(d)** and arabitol in the shoots of unstressed and water-deficit stressed plants in Experiment 2 with the 278/279 clone pair. Statistical significance is indicated as in Figure 1. Error bars are SEM (n = 3).

Levels of different sugar alcohols, including myo-inositol, mannitol, sorbitol, arabitol, galactinol, and chiro-inositol, were quantified in clone pair 278/279. Significant increases in myo-inositol were observed (days 2 and 3) in response to water deficit, but there was no significant effect of endophyte (Figure 6a, Table 2). Mannitol, a fungal metabolite, was undetectable in E- plants at most time points, but increased significantly in E+ plants at day 3 after withholding water, compared to E+ water controls (Figure 6b, Table 2). Sorbitol was found in both E+ and E- plants, and water deficit and endophyte did not influence these levels significantly (Figure 6c, Table 2). Arabitol was not found in either E+ or E- watered controls, but upon water deficit stress, arabitol accumulated with a maximum at day 3 in E+ plants (Figure 6d, Table 2). Chiro-inositol levels were very low, and galactinol levels were not significantly affected by the endophyte or water deficit status (data not shown).

### Amino acids

**Figure 7:**
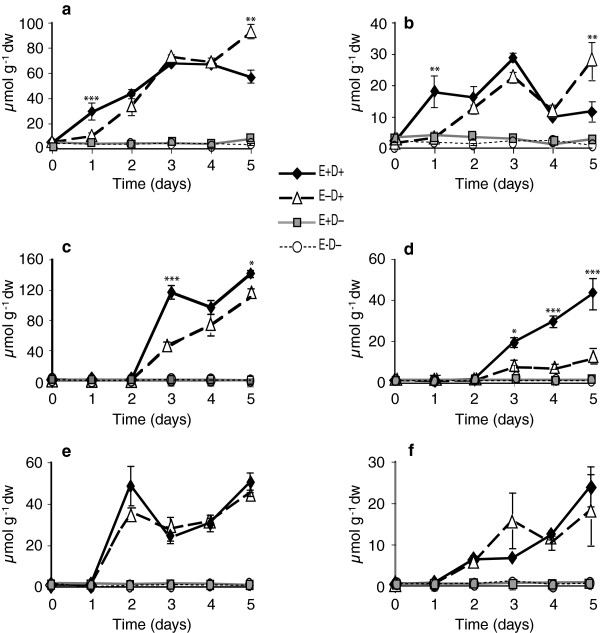
**Proline levels in water-deficit stressed and unstressed shoots and roots of tall fescue. (a** and **b)** Shoots and roots, respectively, of 278/279 clone pair, Experiment 1; **(c** and **d)** shoots and roots, respectively, of 278/279 clone pair, Experiment 2; **(e** and **f)** shoots and roots, respectively, of 4607/4608 clone pair. Abbreviations are as in Figure 2. Statistical significance is indicated as in Figure 1. Error bars are SEM of biological replicates as indicated in Figure 2.

A total of 11 free amino acids were measured in watered controls and stressed shoot and root tissues of both clone pairs. The amino acids, methionine, arginine, ornithine and homoserine, were very low or undetectable. Levels of the amino acids valine, tryptophan, tyrosine, threonine, and phenylalanine were higher in stressed plants from day 2 to day 5 compared to watered controls, but no consistent endophyte effects on these amino-acid levels were observed (Tables 1, 2 and 3). Serine levels did not change due to endophyte or water deficit stress. In the first experiment with the 278/279 clone pair, proline levels in shoots and roots of E+ and E- clones increased under water deficit stress, but not in watered controls (Figure 7a and b, Table 1). At day 1 of withholding water, levels of proline increased approximately 6-fold in E+ shoots and roots, whereas comparable increases in E- plants were not observed till day 2. Thus, levels of proline at day 1 were significantly greater in E+ than in E- plants (Figure 7a and b). On day 2 of water deficit, and thereafter, there were no significant differences in the levels of proline between E+ and E- plants until day 4. However, levels in the treated clones remained approximately 13-15-fold higher than in watered controls. In this experiment, the elevated levels of proline were accompanied by a slight decrease in glutamine levels (data not shown), but the total levels of proline, glutamine, and glutamic acid, which are metabolically interrelated, were higher in the stressed tissues. There were no significant differences in asparagine levels between E+ and E- plants upon water deficit (Table 1).

In the second experiment with clone pair 278/279, increases in amino acid levels started from day 3 after withholding water. At that time point, proline levels in E+ clones were significantly higher than in E- clones both in shoots (Figure 7c) and roots (Figure 7d).

**Figure 8:**
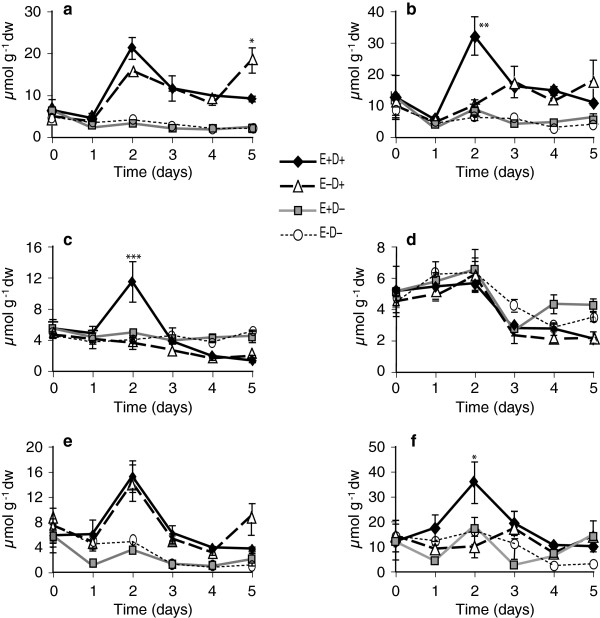
**Some of the metabolite levels in 4607/4608 clone pair shoots of water-deficit stressed and unstressed plants. (a)** glutamine in the shoot **(b)** glutamine in the root **(c)** glutamic acid in the shoot **(d)** glutamic acid in the root **(e)** asparagine in the shoot **(f)** asparagine in the root. Abbreviations are as in Figure 2. Statistical significance is indicated as in Figure 1. Error bars are SEM (n = 3).

In the experiment with clone pair 4607/4608, proline levels increased in shoots of both E+ and E- plants by day 2 of withholding water, but endophyte effect was not significant (Figure 7e, Table 3). However, levels of glutamine and glutamic acid, which are metabolically linked to proline, were higher at day 2 after water deficit (Figure 8a and c); and glutamic acid was significantly higher in E+ compared to E- shoots (Figure 8c). Similarly, in stressed roots, proline levels did not significantly differ between E+ and E- (Figure 7f, Table 3), but at day 2 glutamine reached approximately 3-fold higher levels in E+ roots compared to E- stressed roots and to watered controls (Figure 8b). Asparagine levels increased in shoots by day 2 of withholding water, but were not significantly different between E+ and E- shoots (Figure 8e, Table 3). However, in roots, asparagine levels were significantly higher at day 2 in E+ compared to E- clones (Figure 8f). Overall, in this genotype, the endophyte effects on metabolites were evident especially in roots within two days of withholding water.

### Loline alkaloids

**Figure 9:**
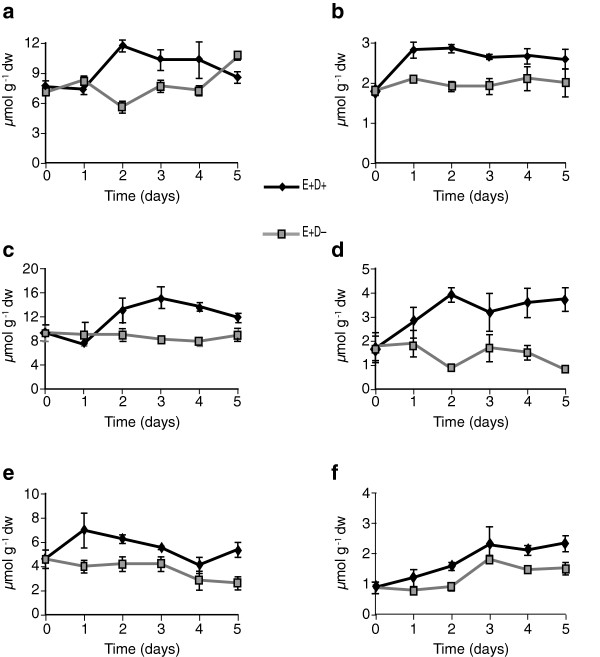
**Loline levels in water-deficit stressed and unstressed shoots and roots of tall fescue. (a** and **b)** Shoots and roots, respectively, of 278/279 clone pair, Experiment 1; **(c** and **d)** shoots and roots, respectively, of 278/279 clone pair, Experiment 2; **(e** and **f)** shoots and roots, respectively, of 4607/4608 clone pair. Abbreviations are as in Figure 2. Statistical significance is indicated as in Figure 1. Error bars are SEM of biological replicates as indicated in Figure 2.

Lolines are the most abundant alkaloids produced by *N. coenophialum* in tall fescue, where the major forms are *N*-formylloline and *N*-acetylloline, although *N*-methylloline and *N*-acetylnorloline are also detected. In Experiment 1 with clone pair 278/279, total loline alkaloid levels in E+ shoot samples were higher in stressed clones from day 2 to day 4 of withholding water (Figure 9a, Table 1). Lower amounts of lolines were detected in root samples compared to shoot samples (Figure 9b). As expected, lolines were undetectable in E- root and shoot samples. In the second experiment with same clone pair, lolines increased in stressed shoots and were significantly different from watered controls by day 3 (Figure 9c and d, Table 2). Loline levels in clone pair 4607/4608 showed similar trends (Figure 9e and f, Table 3).

## Discussion

We assessed plant survival and differences in metabolite accumulation in two tall fescue clone pairs with (E+) or without (E-) symbiotic *Neotyphodium coenophialum* over a time course of water deficit stress, and observed that E+ plants recovered significantly better than E- plants after 2–3 days of withholding water. Simultaneously, the E+ plants consistently accumulated more free sugars, sugar alcohols and amino acids early during the onset of stress, compared to E- plants. The fungal-specific metabolites, mannitol and loline alkaloids, also increased in this time period. The higher metabolite levels in E+ compared to E- plants over the time course of withholding water consistently occurred within one day prior to a significant endophyte effect on plant recovery, strongly suggesting that free sugars, polyols, amino acids, and fungal metabolites play roles in endophyte-enhanced tolerance to water deficit. The production or release of these substances may lead to osmotic adjustment [20,36], and help maintain integrity of cellular enzymes, proteins, nucleic acids and membranes [37], or protect against reactive oxygen species (ROS) [38,39].

The accumulation of soluble sugars is strongly correlated with drought tolerance in plants [40]. These sugars affect osmotic adjustment, which is considered an important mechanism to allow maintenance of water uptake and cell turgor under stress conditions [41]. Furthermore, hydroxyl groups of sugars and polyols can interact with proteins and membranes to prevent denaturation and help avoid the crystallization of cytoplasm under low-water stress [42,43]. In addition, these sugars have been shown to be important regulatory molecules in different signaling pathways [22,44], helping to maintain redox balance, and acting as reactive oxygen scavengers [45,46]. In general, endophytic fungi are similar to plant pathogenic fungi in possessing glucan hydrolase-32 (GH32 invertase) enzymes that convert sucrose into glucose and fructose for catabolism [47]. Fungal invertase activity and presence of invertase gene transcripts have been reported in some of the grass endophytes [48,49], so under the conditions imposed in our study, fungal enzymes may play at least a partial role in the observed increases in these free sugars.

Mannitol and arabitol are common polyols in fungi, and have been observed to accumulate in plants during infection [50]. We found that both polyols increased in response to water deficit in the E+ tall fescue clones. Our results, are in agreement with Richardson et al. [26] who reported mannitol in E+ tall fescue plants, although they did not see an effect on the mannitol levels when the plants were osmotically stressed with polyethylene glycol. Arabitol accumulated essentially only under stress (Figure 6) conditions [26]. Most plants do not normally contain mannitol, with some salt tolerant species, such as celery, as exceptions [51]. Note that the very low levels of mannitol in some E- plants was likely due to the presence of commensal fungi on the plants, since the plants were not grown axenically. Plants engineered to produce mannitol have shown increased tolerances to drought, salt, and temperature stresses [52-55], so mannitol in the E+ plants may have contributed to their tolerance of water deficit stress.

The non-reducing disaccharide, trehalose, is an important osmoprotectant and storage carbohydrate in many organisms. In plants, the trehalose pathway is ubiquitous and indispensible, but with a few exceptions, such as in resurrection plants, trehalose typically does not accumulate to high levels, possibly due to trehalase-catalyzed cleavage to glucose. Significant increases in trehalose accumulation have been accomplished thorough transgenic approaches, and shown to protect plants from drought and salt stresses [56-59]. However, the overproduction or accumulation of high levels of trehalose is also observed to cause growth aberrations in some of the transgenic experiments [60-63]. In our studies, we observed increased levels of trehalose after 3 days of withholding water, with significantly higher levels in E+ plants. Although the overall levels of trehalose observed in the E+ and E- plants were very low compared to the other soluble sugars and polyols, the observed spike in trehalose accumulation during stress, and differences between E+ and E- plants in trehalose levels suggest a possible functional role. While it is possible that the low trehalose levels observed in these plants could function in stress tolerance [64], it seems more likely that the trehalose accumulation is associated with the signaling/regulation role that has been documented [65-70].

Water deficit has been shown to increase levels of ROS, so an important role of accumulated metabolites appears to be scavenging or detoxifying ROS [45,71,72]. Production of phenolics, carbohydrates, mannitol, and proline with antioxidant capacity protects plants from oxidative stress under water-deficit conditions. As reviewed by White and Torres [73], symbiotic plants are protected from different abiotic and biotic stresses by production of these antioxidants.

The timing of metabolite changes was also highly suggestive of their roles in endophyte-enhanced stress tolerance. In all three experiments we observed endophyte-enhanced increases in certain sugars, sugar alcohols and amino acids one day before observing the significant endophyte effect on recovery of the stressed plants. Interestingly, endophyte effects on levels of most metabolites were brief, since levels of these metabolites in E+ plants decreased or plateaued over the following days to levels similar to those in E- plants. In addition to enhancing osmotic adjustment, it is also possible that these accumulated solutes provided energy, carbon and nitrogen for the survival of meristematic regions, and helped in regrowth of the plant after the water deficit was alleviated.

Levels of several amino acids have been shown to increase in drought stressed plants [74]. In our experiments, the levels of proline, threonine, tryptophan, phenylalanine, tyrosine, and valine increased upon water deficit stress. In addition, proline was found to be consistently higher in both shoots and roots of E+ stressed plants than in E- stressed plants. A correlation between free proline accumulation and the performance of crops in the field at low water availability suggests that its accumulation is a drought stress adaptive response that enhances survival [75]. Proline may serve as an osmoregulator [74] and also as a ROS scavenger [76].

Loline alkaloids are protective secondary metabolites produced by the endophyte in tall fescue and other cool season grasses [77,78]. We observed increased loline alkaloid levels in response to water deficit stress in both clone pairs. Lolines are derived from proline and aspartate [79]. Conceivably, proline is depleted by loline production [80], but since no differences in proline levels were observed between E+ and E- plants in unstressed conditions, proline levels were apparently adjusted in response to loline alkaloid synthesis. In the first experiment with clone pair 278/279 total proline and loline levels in E+ plants were higher even at day 2 after withholding water compared to E- plants, though levels of proline (and the metabolically closely related amino acids, glutamic acid and glutamine) were not different in between E+ and E- plants at day 2. It is possible that the proline is converted to loline in the E+ plants, thus maintaining an apparent equal proline level as that of E- plants. However, in the other two experiments, the levels of proline in stressed tissues were far higher compared to amounts of lolines that accumulated in those tissues. Although water deficit has been reported to increase loline alkaloid levels in leaf tissues of some tall fescue accessions [81], a direct role of loline alkaloids on water stress tolerance has not yet been demonstrated.

Differences in the timing of metabolite accumulation were observed between two experiments with the same clone pair (278/279), with metabolite peaks at day 1 in Experiment 1 and the corresponding peaks occurring at day 2 or 3 in Experiment 2. This difference may be because of weather and greenhouse conditions that differed between these experiments. Specifically, day 1 of Experiment 2 was accompanied with thunderstorms and heavily overcast skies, resulting in lower photoactive radiation compared to day 1 of Experiment 1 (see Additional file 1, panel b), apparently delaying the onset of drought stress as evidenced by the tiller recovery curves (see Figure 1a and b). Similarly the observed metabolite differences between the experiments with different clone pairs could be due to plant genotype effects. Nevertheless, it was clear that, in our experiments the endophyte in tall fescue sped up plant responses to water deficit by earlier and faster accumulation of metabolites compared to uninfected tall fescue plants. Similar results have been reported in bacterial endophyte-plant systems. Bacterial endophyte enhances cold tolerance of grapevine plants by altering sugar metabolism and photosynthesis [82], and with higher and faster accumulation of stress related gene transcripts and metabolites [83].

Rasmussen et al. [84] have conducted comprehensive metabolomic studies in the related grass, *Lolium perenne* (perennial ryegrass), and have shown significant effects of the endophyte, *Neotyphodium lolii,* on primary and secondary metabolism of that grass. The need for more research to identify robust metabolic traits and pathways relating to drought tolerance in forage grasses through integration of metabolomic and transcriptomic data have been emphasized in reviews [85]. From our study it was evident that endophyte can affect tall fescue plant metabolism, in response to water deficit stress. Analyzing these endophyte effects on host plants at the molecular genetic level by transcriptome profiling is another approach, that we will be exploring further to help elucidate the mechanisms of endophyte-enhanced plant growth and survival under water deficit conditions.

## Conclusions

In conclusion, enabling the plant cells to sense and respond quickly to surrounding environmental signals or stresses is important for their metabolic and developmental adjustments, and these responses may be enhanced due either to primary or secondary metabolite signals [86,87]. As we observed in the tall fescue clone pairs, symbiotic fungi in the infected plants may have induced, or rapidly activated, the plant biochemical reactions to accumulate the metabolites early in stress conditions, and this may be one of the ways that the presence of the endophyte helps mitigate the effects of, and enhance recovery from, water deficit stress. The results presented here demonstrate that symbiosis with endophytes can significantly enhance recovery of host plants from water deficit stress, and the effect corresponds in timing with accumulation of organic solutes that may serve as osmolytes and cellular protectants in leaves and roots.

## Competing interests

The authors declare that they have no competing interests.

## Authors’ contributions

PN designed and performed all experiments, and drafted the manuscript. RDD helped design experiments, generated and provided clone pair 4607/4608, and helped draft the manuscript. CLW performed statistical analyses. CWB generated and provided clone pair 278/279, and helped draft the manuscript. CLS devised and supervised experiments and helped draft the manuscript. All authors read and approved the final manuscript.

## Supplementary Material

Additional file 1 **Photoactive radiation (PAR) at the sampling period.** (a) clone pair 278/279, Experiment 1; (b) clone pair 278/279, Experiment 2; (c) clone pair 4607/4608. Click here for file

Additional file 2 **Total amounts of glucose, fructose, sucrose (GFS) in water-deficit stressed and unstressed plants of tall fescue clone pairs.** (a and b) Shoots and roots, respectively, of clone pair 278/279, Experiment 1; (c and d) shoots and roots, respectively, of clone pair 278/279, Experiment 2; (e and f) shoots and roots, respectively, of clone pair 4607/4608. Click here for file
