# Supplementary figures and images for: Tall fescue endophyte effects on tolerance to water-deficit stress
Source: BMC Plant Biol. 2013 Sep 9;13:127. doi: 10.1186/1471-2229-13-127 (PMC3848598; doi:10.1186/1471-2229-13-127)

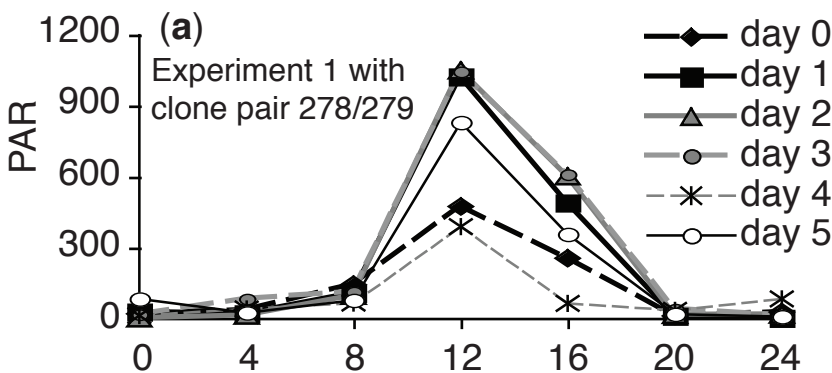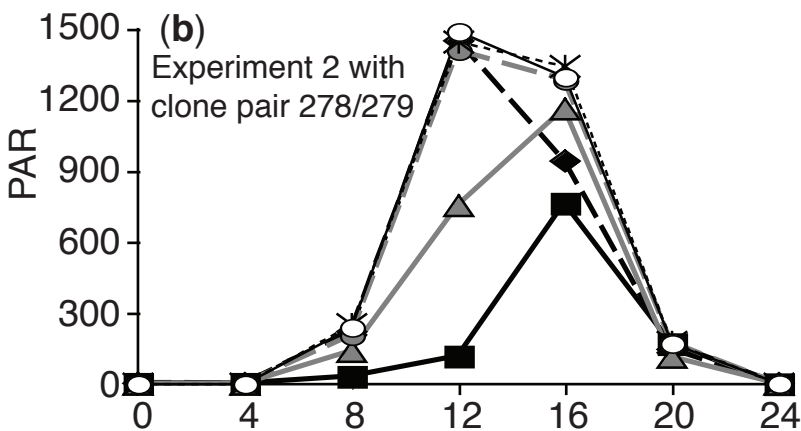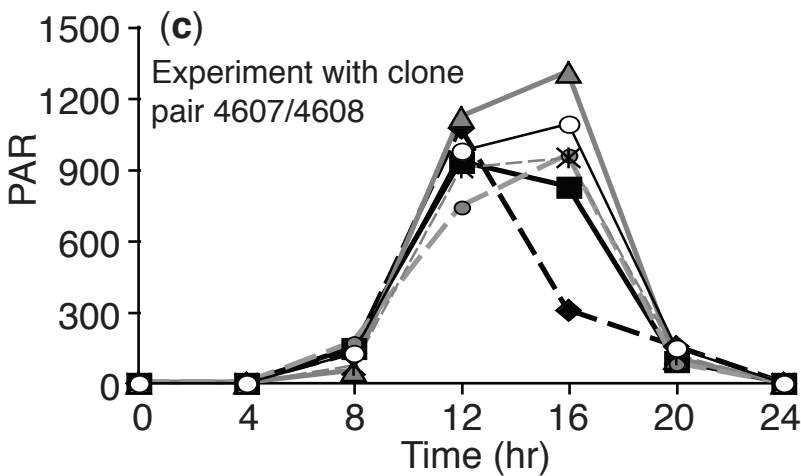

Supplement: Additional file 1 — Photoactive radiation (PAR) at the sampling period. (a) clone pair 278/279, Experiment 1; (b) clone pair 278/279, Experiment 2; (c) clone pair 4607/4608. [file 1471-2229-13-127-S1.pdf]

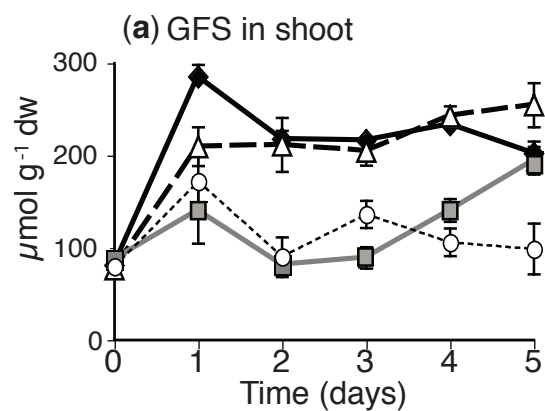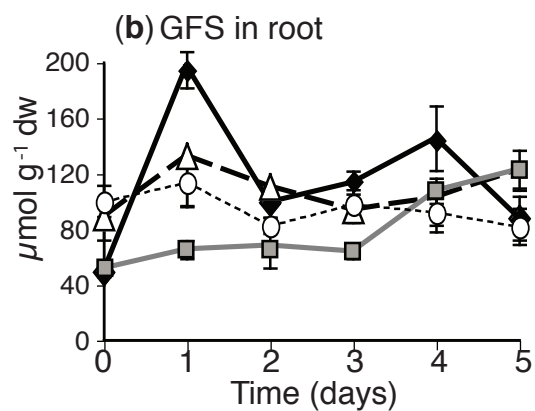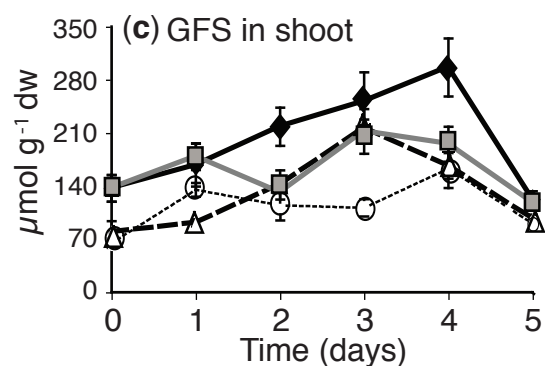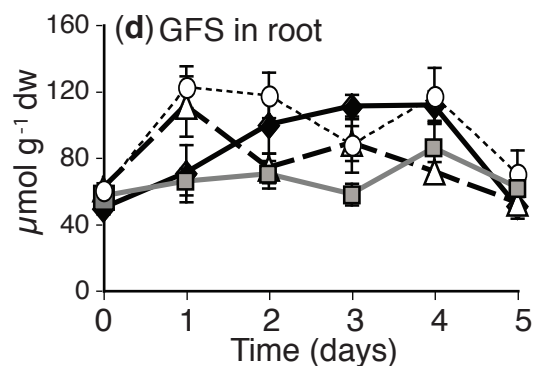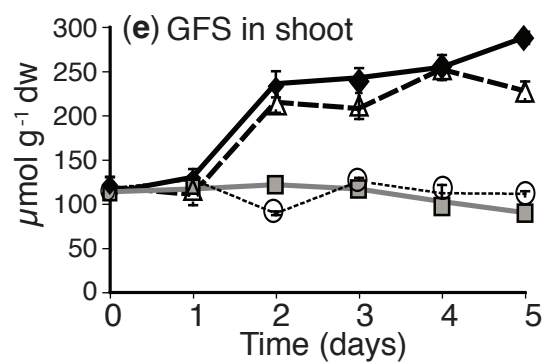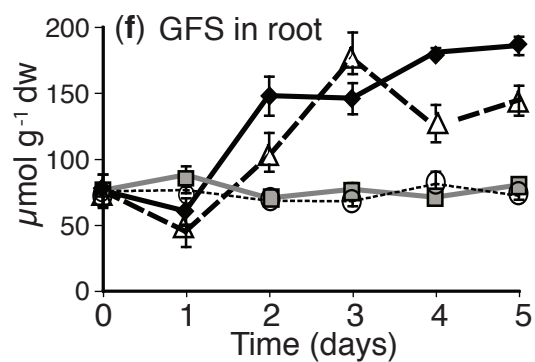

Supplement: Additional file 2 — Total amounts of glucose, fructose, sucrose (GFS) in water-deficit stressed and unstressed plants of tall fescue clone pairs. (a and b) Shoots and roots, respectively, of clone pair 278/279, Experiment 1; (c and d) shoots and roots, respectively, of clone pair 278/279, Experiment 2; (e and f) shoots and roots, respectively, of clone pair 4607/4608. [file 1471-2229-13-127-S2.pdf]
